# Supplementary material for: Contextualizing the think crisis-think female stereotype in explaining the glass cliff: Gendered traits, gender, and type of crisis
Source: PLoS One. 2021 Mar 2;16(3):e0246576. doi: 10.1371/journal.pone.0246576 (PMC7924740; doi:10.1371/journal.pone.0246576)
Supplement: S1 Appendix — (DOCX) [file pone.0246576.s001.docx]

### S1 Appendix.

### Study 1 Manipulation materials

**A1a Table. Three crisis type manipulations used in Study 1 (original Spanish version).** Participants were randomly allocated to one of these texts.

| Relational crisis | Financial crisis | No crisis |
| --- | --- | --- |
| Ahora, por favor párate un momento a pensar cómo podría ser tu vida dentro de unos 10 o 15 años. Imagina que estás trabajando como empleado/a de una empresa que ha sufrido una crisis bastante seria. Concretamente, piensa que la empresa está atravesando problemas internos relacionados con el hecho de que las personas no trabajan bien de manera conjunta. Este problema de armonía ha dañado seriamente la motivación de los/as empleados/as y ha creado una atmósfera negativa, lo cual ha hecho que se produzca un significativo descenso en los beneficios de la empresa.  Incluso aunque este escenario no describa tu futuro esperado realmente, por favor dedica un par de minutos a escribir un breve párrafo sobre qué retos tendrías en tu día a día trabajando en esta empresa. | Ahora, por favor párate un momento a pensar cómo podría ser tu vida dentro de unos 10 o 15 años. Imagina que estás trabajando como empleado/a de una empresa que ha sufrido una crisis bastante seria. Concretamente, piensa que la empresa en la que trabajas está en la situación de que otras empresas del mismo sector han expandido su control sobre el mercado en situaciones difíciles ofreciendo mejores productos y servicios. La empresa ha salido perdiendo respecto a la competencia, que ha introducido nuevos productos de forma más agresiva. Además, las previsiones financieras no han sido adecuadas, lo cual en conjunto ha hecho que se produzca un significativo descenso en los beneficios de la empresa.  Incluso aunque este escenario no describa tu futuro esperado realmente, por favor dedica un par de minutos a escribir un breve párrafo sobre qué retos tendrías en tu día a día trabajando en esta empresa. | Ahora, por favor párate un momento a pensar cómo podría ser tu vida dentro de unos 10 o 15 años. Imagina que estás trabajando como empleado/a de una empresa.  Incluso aunque este escenario no describa tu futuro esperado realmente, por favor dedica un par de minutos a escribir un breve párrafo sobre qué retos tendrías en tu día a día trabajando en esta empresa. |

**A1b Table. Three crisis type manipulations used in Study 1 (English translation).**

| Relational crisis | Financial crisis | No crisis |
| --- | --- | --- |
| Now, please take a moment to think what your life might be like in 10-15 years. Imagine that you are working as an employee of a company that has suffered a serious crisis. Specifically, think that the company is experiencing internal problems related to the fact that people do not work well together. This harmony problem has seriously damaged the motivation of employees and created a negative atmosphere, which has caused a significant decrease in the profits of the company.  Even if this scenario does not really describe your expected future, please take a couple of minutes to write a short paragraph about what challenges you would have in your day to day working at this company. | Now, please take a moment to think what your life might be like in 10-15 years. Imagine that you are working as an employee of a company that has suffered a serious crisis. Specifically, think that the company is in the situation that other companies in the same sector have expanded their control over the market in difficult situations by offering better products and services. The company has lost out to the competition, which has introduced new products more aggressively. Moreover, the financial forecasts have not been adequate, which has caused a significant decrease in the profits of the company.  Even if this scenario does not really describe your expected future, please take a couple of minutes to write a short paragraph about what challenges you would have in your day to day working at this company. | Now, please take a moment to think what your life might be like in 10-15 years. Imagine that you are working as an employee of a company.  Even if this scenario does not really describe your expected future, please take a couple of minutes to write a short paragraph about what challenges you would have in your day to day working at this company. |

**A2 Table. The five candidate descriptions of Study 1.** Participants saw all five candidates from which they chose one.

| **Candidate gender, gendered traits** | **Name of candidate** | **Description of candidate (original Spanish)** | **Description of candidate (english translation)** |
| --- | --- | --- | --- |
| **Male, extreme agentic** | Mikel | Tiene una gran capacidad para afrontar dificultades. Es de carácter activo y muestra una gran auto-confianza en todo lo que hace. Su carácter independiente le permite tomar decisiones de manera autónoma y decidida. | He has a great ability to cope with difficulties. He is active in character and shows great self-confidence in everything he does. His independent nature allows him to make decisions autonomously and decisively. |
| **Male, agentic** | Roverto | Es competitivo y ambicioso en sus metas. Le gusta estar al mando en la mayoría de situaciones y destaca por ser una persona firme, dura e incluso algo autoritaria. Es capaz de actuar sin miramientos cuando las circunstancias lo requieren. | He is competitive and ambitious in his goals. He likes to have the control in most situations and stands out for being a firm, tough and even somewhat authoritarian person. He is capable of acting ruthlessly when circumstances require. |
| **Female, agentic** | Isabel | Capacidad para tomar la iniciativa sin esperar a tener la aprobación de los demás. Tiene una gran confianza en sí misma y muestra una alta orientación al logro. Es activa, luchadora y rara vez se deja abatir por los obstáculos. | Ability to take initiative without waiting for the approval of others. She has great self-confidence and shows high achievement orientation. She is active, determined, and rarely gets knocked down by obstacles. |
| **Male, communal** | Janvier | Mantiene buenas relaciones con la gente que le rodea y es agradable en su trato con los demás. Es capaz de dedicar tiempo a escuchar las necesidades ajenas y se suele comportar de manera considerada y amable. | He maintains good relationships with the people around him and is kind in his relationships with others. He is able to spend time listening to the needs of others and usually behaves in a considerate and friendly manner. |
| **Female, communal** | Maria | De carácter amable y abierto. Tiene una gran capacidad de escucha y tiende a mostrar comprensión por las personas. Tiene una gran habilidad para tratar con la gente y le gusta ayudar a los demás cuando está en su mano. | Kind and open-minded. She has a great ability to listen and tends to be understanding with people. She has a great ability to related to people and likes to help others when it is possible. |

### Study 2 Manipulation materials

**A1 Fig. Three crisis type articles of Study 2.** Participants were randomly allocated to one of these texts.

| Relational crisis | Financial crisis | No crisis |
| --- | --- | --- |
| 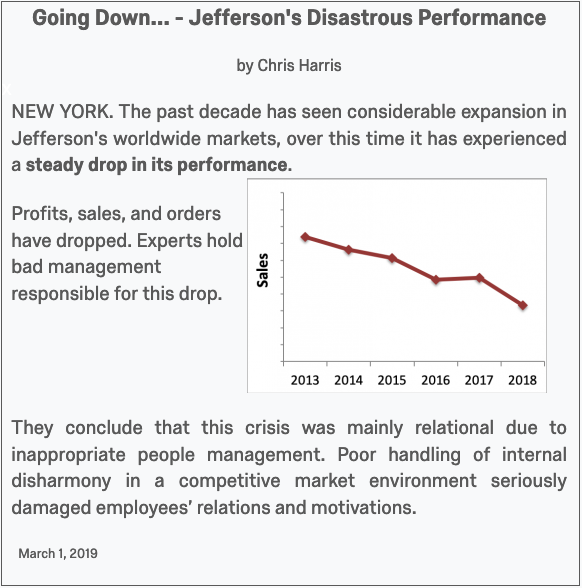 | 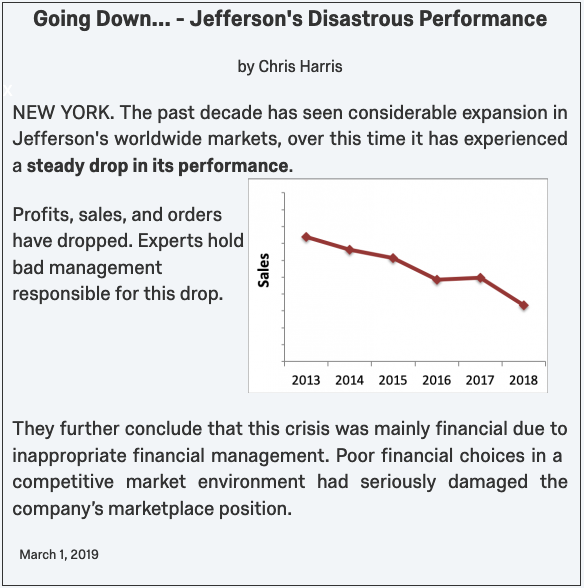 | 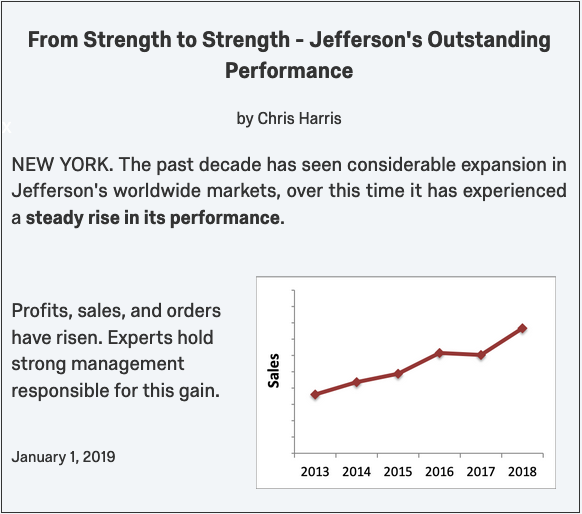 |

**A3 Table. Four candidate descriptions of Study 2.** Participants saw all four candidates from which they chose one. Note only one out of two counterbalanced versions (A and B) was randomly displayed to each participant.

| **Candidate gender, gendered traits** | **Candidate name** | **Version A** | **Version B** |
| --- | --- | --- | --- |
| **Male, agentic** | Alan Murphy | He has a great capacity to face difficulties. He is active and shows great self-confidence in everything he does. His independent nature allows him to make decisions autonomously and decisively. | He has the ability to take initiative without waiting for others' approval. He has great confidence in himself and shows a high achievement-orientation. He is active, competitive and rarely lets himself be overcome by obstacles. |
| **Female, agentic** | Claire Brown | She has the ability to take initiative without waiting for others' approval. She has great confidence in herself and shows a high achievement-orientation. She is active, competitive and rarely lets herself be overcome by obstacles. | She has a great capacity to face difficulties. She is active and shows great self-confidence in everything she does. Her independent nature allows her to make decisions autonomously and decisively. |
| **Male, communal** | Philip Hawker | He maintains good relationships with the people around him and he is pleasant when dealing with others. He is able to spend time listening to the needs of others and he is usually a considerate and kind person. | He has a kind and open character. He has a great capacity for listening and tends to be understanding with others. He has a great ability to deal with people and likes to help others when it is in his hand. |
| **Female, communal** | Mary Jones | She has a kind and open character. She has a great capacity for listening and tends to be understanding with others. She has a great ability to deal with people and likes to help others when it is in her hand. | She maintains good relationships with the people around her and she is pleasant when dealing with others. She is able to spend time listening to the needs of others and she is usually a considerate and kind person. |

### Study 3 Manipulation materials

**A2a Fig. Three versions of “Relational crisis” articles of Study 3.** Each participant saw three companies affected by three different crisis types from which they had to choose one. Note that only one randomized combination of three companies of different names (Campbell, Jefferson and Morton) affected by three different crisis types was shown to each participant (A2a Fig. to A2c Fig.).

| **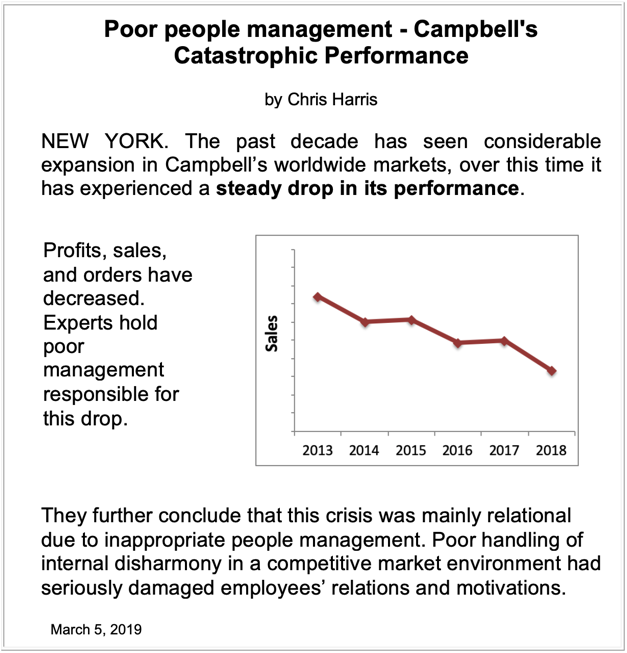** | **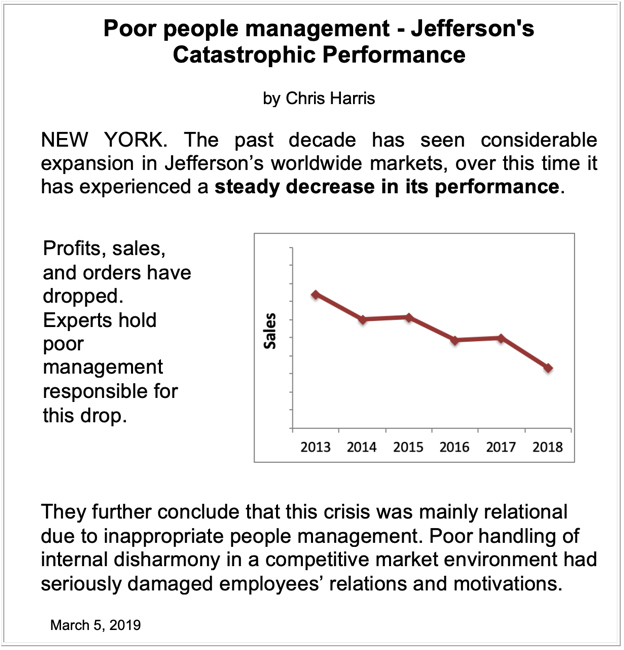** | **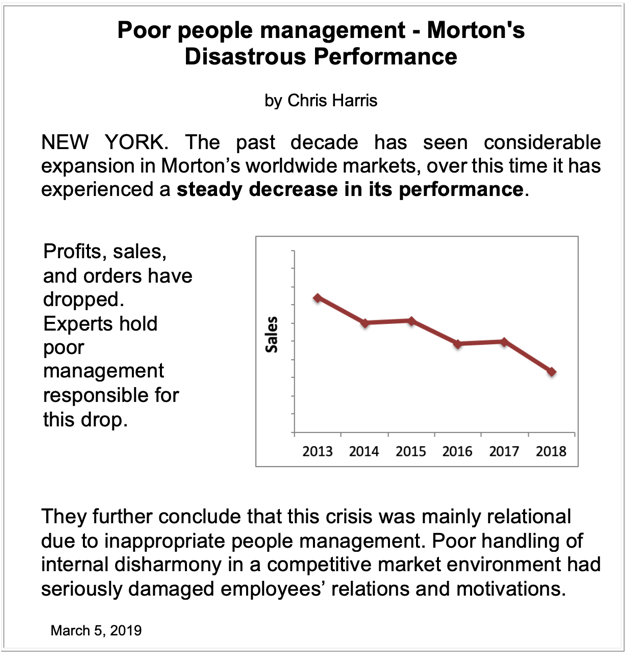** |
| --- | --- | --- |

**A2b Fig. Three versions of “Financial crisis” articles of Study 3.**

| 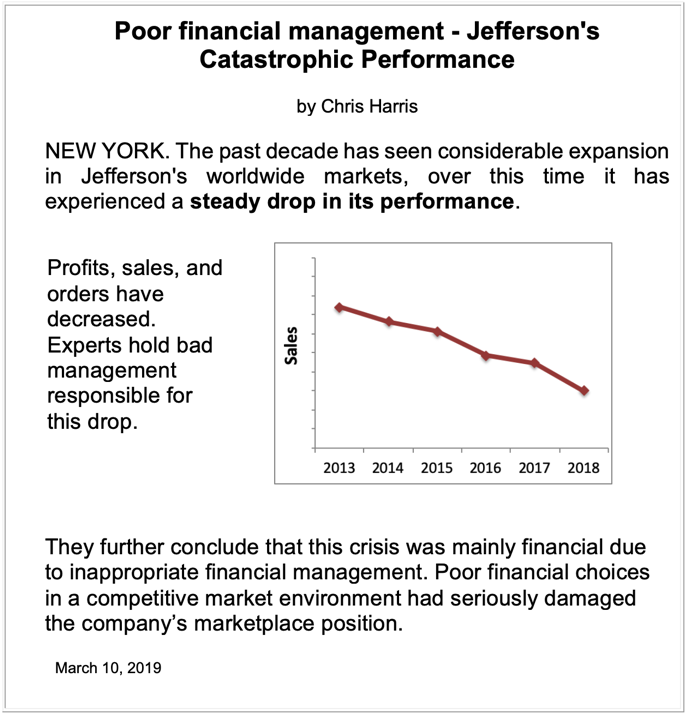 | 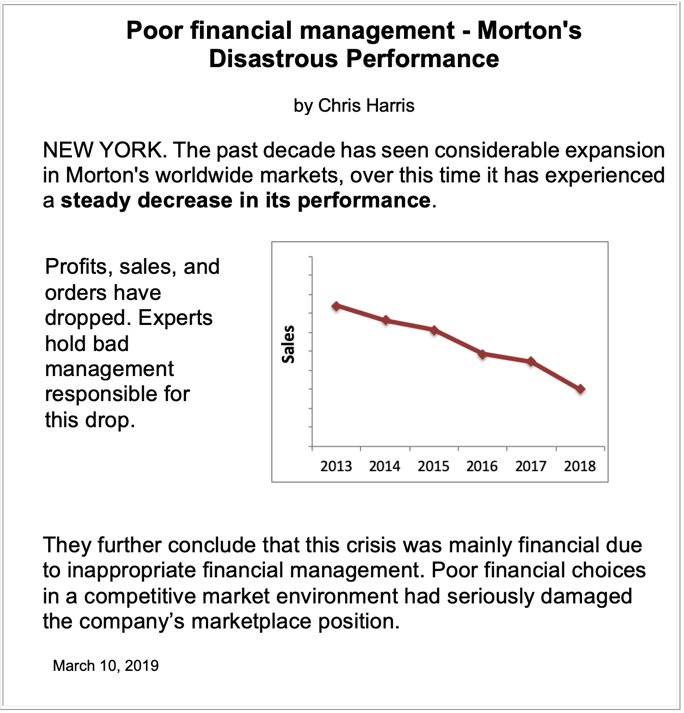 | 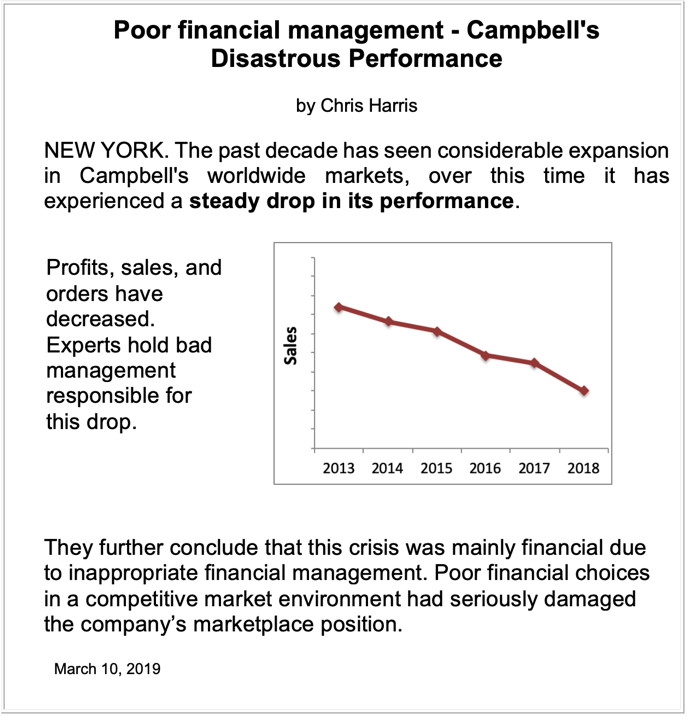 |
| --- | --- | --- |

| 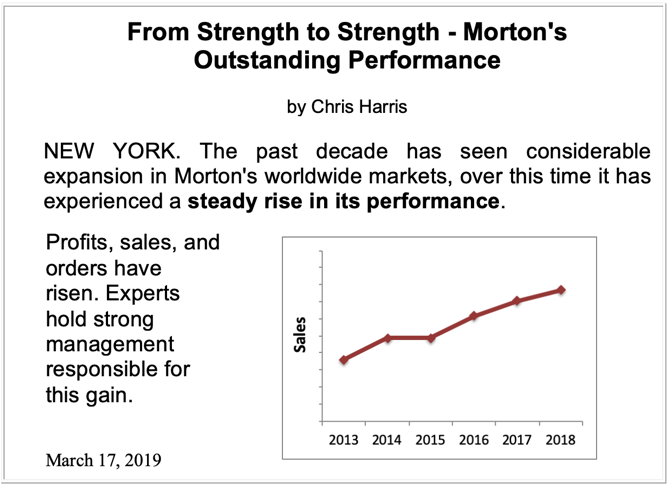 | 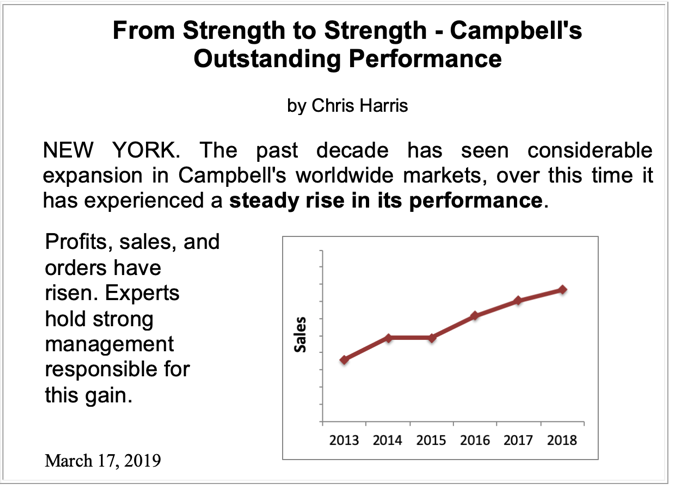 | 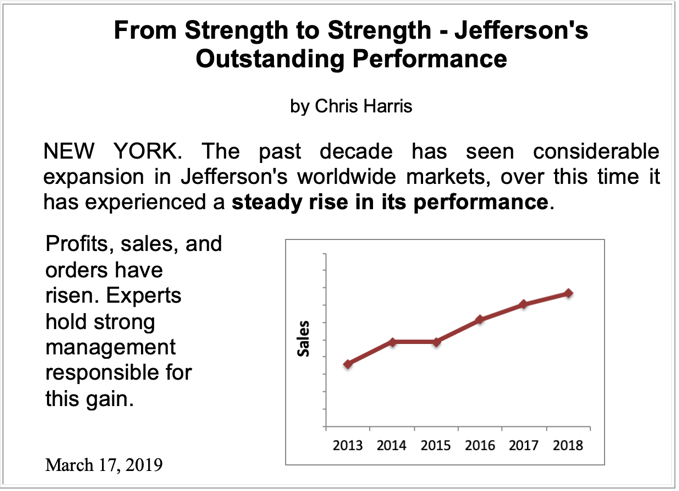 |
| --- | --- | --- |

**A2c Fig. Three versions of “No crisis” articles of Study 3.**

**A4 Table. Four candidate descriptions of Study 3.** Each participant saw only one of the four candidate descriptions. Note that each gender-gendered traits combination had two counterbalanced and randomized versions (A and B).

| **Candidate gendered traits** | **Female candidate** | **Male candidate** |
| --- | --- | --- |
| **Agentic candidate**  **Version A** | Claire Jones has a great capacity to face difficulties. She is active and shows great self-confidence in everything she does. Her independent nature allows her to make decisions autonomously and decisively. | Alan Jones has a great capacity to face difficulties. He is active and shows great self-confidence in everything he does. His independent nature allows him to make decisions autonomously and decisively. |
| **Agentic candidate**  **Version B** | Claire Jones has the ability to take initiative without waiting for others' approval. She has great confidence in herself and shows a high achievement-orientation. She is active, competitive and rarely lets herself be overcome by obstacles. | Alan Jones has the ability to take initiative without waiting for others' approval. He has great confidence in himself and shows a high achievement-orientation. He is active, competitive and rarely lets himself be overcome by obstacles. |
| **Communal candidate Version A** | Claire Jones maintains good relationships with the people around her and she is pleasant when dealing with others. She is able to spend time listening to the needs of others and she is usually a considerate and kind person. | Alan Jones maintains good relationships with the people around him and he is pleasant when dealing with others. He is able to spend time listening to the needs of others and he is usually a considerate and kind person. |
| **Communal candidate**  **Version B** | Claire Jones has a kind and open character. She has a great capacity for listening and tends to be understanding with others. She has a great ability to deal with people and likes to help others when it is in her hand. | Alan Jones has a kind and open character. He has a great capacity for listening and tends to be understanding with others. He has a great ability to deal with people and likes to help others when it is in his hand. |
