# Supplementary material for: Contextualizing the think crisis-think female stereotype in explaining the glass cliff: Gendered traits, gender, and type of crisis
Source: PLoS One. 2021 Mar 2;16(3):e0246576. doi: 10.1371/journal.pone.0246576 (PMC7924740; doi:10.1371/journal.pone.0246576)
Supplement: S1 File — (DOCX) [file pone.0246576.s002.docx]

# S1 File. Supporting information

Article title: Contextualizing the think crisis-think female stereotype in explaining the glass cliff: Gendered traits, gender, and type of crisis

For reasons of transparency, we present in the Supporting information, all measures from the original questionnaires which were not reported in the main manuscript, as well as exploratory analyses. It further contains more details on a participant gender effect found in Study 1, and factor analyses of scales used in the main manuscript.

## Study 1

### Preferences for candidates

**S1 Table. Underpowered explorative post-hoc comparisons of candidate choices per crisis condition (Study 1).**

|  | Relational vs Financial crisis | Relational vs No crisis | Financial vs No crisis |
| --- | --- | --- | --- |
| Candidate gendered traits | *p* < .001 | *p* = .067 | *p* = .149 |
| Candidate gender | *p* = .246 | *p* = .034 | *p* = .293 |

### Leadership behaviors and traits

In addition to the communal dimension presented in the main article, four task-oriented items from the “producer” dimension (Focus on results for the organization; Ensure the organization delivers on stated goals; Push the organization to meet objectives; Emphasize organization’s achievement of stated purposes; α = .68; *M =* 4.83, *SD* = 0.69) measured *agentic behavior*, and eight items agentic traits (e.g., *independent, active, decisive, never gives up, competitive, self-reliant, stands up under pressure*, *feels superior*). A Promax principal axing factoring factor analysis on the communal and agentic traits which revealed three factors, with the first (30.06% of variance explained) containing the communal traits, the second (14.82%) and third (8.92%) agentic traits. Based on reliability analysis of the agentic items, one item “feels superior” had a negative corrected item total correlation with the scale of *r* = -.34. Its exclusion from the scale improved the Cronbach alpha from .55 to .67. The item “feels superior” was thus dropped from the final 7-item agentic trait dimension (α = .67; *M =* 4.91, *SD* = 0.61). Further, eight negative filler items were presented (*arrogant, boastful, self-centered, greedy, dictatorial, cynical, unscrupulous, hostile*). Table S1 presents all estimates for the communion and agency dimension for the purpose of descriptive comparison of the dimensions.

**S2 Table*.* Estimates of leadership behavior and traits evaluations (6-point scale) in each of the three crisis conditions (Study 1).**

|  |  | Estimated Means (SE) | | | | |  | |
| --- | --- | --- | --- | --- | --- | --- | --- | --- |
| Leadership | Dimension | Relational crisis | | Financial crisis | | | No crisis | |
| Behavior | Communion | 4.87 | (0.08) | 4.56 | (0.08) | 4.85 (0.09) | |  |
|  | Agency | 4.69 | (0.06) | 4.87 | (0.06) | 4.94 (0.07) | |  |
| Traits | Communion | 4.46 | (0.07) | 4.51 | (0.07) | 4.68 (0.08) | |  |
|  | Agency | 4.82 | (0.06) | 4. 89 | (0.06) | 4.96 (0.06) | |  |

### Mediation

We did not predict that the effect of crisis type on candidate gender would be mediated by relevance ratings of communal leadership behavior. However, we explored a mediation model 4, using the same model as reported for gendered traits in the main article with the exception of the crisis type predictor (1 = relational crisis, 0 financial crisis, -1 = no crisis). There was no support of a mediation (communal behavior: *B* = 0.003, *SE* = 0.02, 95% CI [-0.03, 0.04]).

### Participant gender

When adding participant gender as main effect and in all interactions to the ANOVA on communal leadership behaviors, the effect of crisis type C1 remained, *F* (l, 307) = 8.27, *p* < .004, η_p_^2^ =.03, as well as the main effect of organizational role, *F* (l, 307) = 4.90, *p* = .028, η_p_^2^ =.02. In addition, participant gender moderated the organizational role effect, *F* (l, 307) = 3.45, *p* = .026, η_p_^2^ =.02 and a Participant gender × C2 interaction was revealed, *F* (l, 307) = 3.94, *p* = .048, η_p_^2^ =.01. All other effects were non-significant (*p*s > .056).

When adding participant gender to the ANOVA on communal traits, the effect of residual contrast C2 remained significant, *F* (l, 307) = 5.16, *p* = .024, η_p_^2^ =.02, and an interaction between participant gender, organizational role and the crisis type C1 was revealed, *F* (l, 307) = 4.74, *p* = .030, η_p_^2^ =.02. We decomposed the effect for each gender but the C1 × Organizational role interaction was neither significant for men nor for women (*p*s > .110).

### Leader suitability

Leader suitability items were presented just after reading the candidate profiles. They served to make participants focus on the evaluation of the candidates as for their usefulness for the company, but also to see how general leader suitability is evaluated as a function of the experimental conditions. Four items assessed suitability on a 6 point scale, 1 *little* to 6 *much*: “S/He would be a good manager for the company, S/He has the necessary skills to move the company forward, S/He has an appropriate profile for the needs of the company, Her/His way of being fits with the profile required by the company” (For all candidates αs = .87, .66, .90, .89, .91; *M*s *=* 2.78, 3.63, 3.43, 3.66, 3.69; *SD*s = 1.37, 1.16 1.05, 1.08 for the male extreme agentic, male agentic, female agentic, male communal, female communal candidate respectively).

A repeated-measures ANOVA was run with crisis type and organizational role as between-participant factor, and leader suitability of each candidate as within-participants factor, as well as all interactions. There was an effect of candidate, *F* (1240, 1227.55) = 45.29, *p* < .001, η_p_^2^ = .13. Pairwise comparisons showed that the male agentic (*M*_D_ = 0.20, *SE* = 0.07, *p* = .006), the male communal (*M*_D_ = 0.23, *SE* = 0.09, *p* = .009), and the female communal (*M*_D_ = 0.28, *SE* = 0.09, *p* = .001) were evaluated as more suitable than the agentic female candidate who in turn was rated more suitable than the extreme agentic male candidate (*M*_D_ = 0.64, *SE* = 0.07, *p* < .001). No other main or interaction effects were significant (*p*s > .122).

### Candidate characteristics

Ten questions on characteristics of the selected candidates were asked at the end of the survey using bi-polar choice items (e.g., Is the candidate “less than 50 years old” vs “more than 50 years old”).

## Study 2

### Preferences for candidates

**S3 Table*.* Underpowered explorative post-hoc comparisons of candidate choices per crisis condition (Study 2).**

|  | Relational vs Financial crisis | Relational vs No crisis | Financial vs No crisis |
| --- | --- | --- | --- |
| Candidate gendered traits | *p* < .001 | *p* < .001 | *p* = .053 |
| Candidate gender | *p* = .123 | *p* = .046 | *p* = .636 |

### Leadership behaviors and traits

Agentic behavior (α = .93; *M=* 6.07, *SD* = 1.12) and agentic traits (α = .89; *M =* 5.80, *SD* = 1.01) were measured with the same items as in Study 1. Again, a Promax principal axing factoring factor analysis on leadership traits revealed three factors, with the first (42.42% of variance explained) containing the communal traits, the second (18.41%) and third (8.08%) agentic traits. Based on reliability analysis of the agentic items, one item “feels superior” had a *r* = .015 corrected item total correlation with the scale. Its exclusion from the scale improved the Cronbach alpha from .82 to .89. The item “feels superior” was thus dropped from the agentic trait dimension. Table S4 presents all estimates for the communion and agency dimension for the purpose of descriptive comparison of the dimensions.

**S4 Table*.* Estimates of leadership behavior and trait evaluations (7-point scale) in each of the three crisis conditions (Study 2).**

|  |  | Estimated Means (SE) | | | | |  | |
| --- | --- | --- | --- | --- | --- | --- | --- | --- |
| Leadership | Dimension | Relational crisis | | Financial crisis | | | No crisis | |
| Behavior | Communion | 5.60 | (0.12) | 4.81 | (0.12) | 5.09 (0.12) | |  |
|  | Agency | 5.92 | (0.10) | 6.17 | (0.10) | 6.13 (.10) | |  |
| Traits | Communion | 5.48 | (0.09) | 5.05 | (0.09) | 5.15 (0.09) | |  |
|  | Agency | 5.68 | (0.09) | 5.89 | (0.09) | 5.83 (0.09) | |  |

### Leader suitability

*S*uitability was measured with the same items as in Study 1 but on a 7-point scale, 1 *completely disagree* to 7 *completely agree* (For all candidates αs > .93; male agentic *M* *=* 5.33, *SD* = 1.34, female agentic: *M* *=* 5.40, *SD* = 1.38, male communal: *M* *=* 4.98, *SD* = 1.32, female communal: *M* *=* 4.97, *SD* = 1.35).

A repeated-measures ANOVA with crisis type as between-participant factor, and leader suitability according to candidate gender (male versus female) and candidate gendered traits (agentic versus communal) as within-participants factors was performed. The only significant effect was candidate gendered traits, *F* (1, 381) = 17.49, *p* < .001, η_p_^2^ =.04, in the sense that agentic candidates (*M* = 5.38, *SE* = 0.06) were evaluated as more suitable than communal candidates (*M* = 4.97, *SE* = 0.06). Furthermore, a Candidate Gendered Traits × Crisis Type interaction, *F* (2, 381) = 27.36, *p* < .001, η_p_^2^ =.13, revealed that communal candidates were perceived as more suitable in the relational crisis (*M* = 5.37, *SE* = 0.11) than in the other two contexts (financial: *M* = 4.68, *SE* = 0.11; *p* < .001, 95% CIs [0.40, 0.99]; no crisis: *M* = 4.87, *SE* = 0.11; *p* < .001, 95% CIs [0.20, 0.80]) and agentic candidates were perceived as more suitable in the financial crisis and no crisis contexts (financial: *M* = 5.79, *SE* = 0.11; *p* < .001, 95% CIs [0.68, 1.26]; no crisis: *M* = 5.52, *SE* = 0.11; *p* < .001, 95% CIs [0.41, 1.002]) compared to the relational context (*M* = 4.82, *SE* = 0.10). No other main or interaction effects were significant (*p*s > .195).

Leader suitability ratings were more favorable of communal candidates in the relational crisis as compared to the other two contexts which matches the findings on the preference of communal candidates in this context. Moreover, suitability ratings seemed to be distinctive for a candidate’s gendered traits but not as concerns their gender. This further suggests that a candidate’s gendered traits may be more strongly associated with a leader’s effectiveness perceptions as relates to the company situation, whereas candidate gender may rely on other factors.

### Change potential

Previous research used the potential of signaling change and actual change items [1,2]. We presented the 10 previously used items and three newly created ones to evaluate the chosen candidate. Table S3 shows the results of a Promax principal axing factoring factor analysis. In Study 2, we presented items for both concepts in order to further develop these measures. The main theoretical interest concerned signaling change. Actual change was measured by six items in the central column in Table S3 (α = .88, *M* = 5.88, *SD* = 0.90)*.*

**S5 Table. Promax principal axing factoring factor analysis with change potential items.**

|  |  | Factor loadings | | |
| --- | --- | --- | --- | --- |
| Origin | Item | Signaling | Actual1 | Actual 2 |
| 2015 | The fact of appointing this candidate will show that the company wants to change the type of management. | .93 |  |  |
| 2015 | The choice of this candidate symbolizes a visible change for partners and competitors. | .83 |  |  |
| 2018 | The choice of this candidate signals to investors that Jefferson is willing to substantially change things. | .78 |  |  |
| 2018 | Choosing this candidate as CEO for Jefferson symbolizes the start of a new era. | .73 |  |  |
| New | This appointment will have a positive impact on the company's performance on the stock-exchange. |  | -.82 |  |
| New | This candidate is capable of increasing profits, sales and orders. |  | -.78 |  |
| 2015 | This candidate is the most qualified. |  | -.73 |  |
| 2015 | This candidate's leadership style is the most suitable. |  | -.69 |  |
| 2018 | This candidate is capable of changing the company’s position on the market. |  | -.65 |  |
| New | This candidate will have better managerial abilities than the former CEO of Jefferson. | .44 | -.45 |  |
| 2018 | This candidate's ways of leading will be consistent with how things have always been done before. |  |  | .81 |
| 2018 | This candidate is likely to keep the company going on the same track as it was before. |  |  | .78 |
| 2018 | This candidate will do things differently than the routine way of leading. (was actual change in 2018 article) | .74 |  |  |

Only items in black were retained for scale construction. The “origin” column indicates whether items are from [3], [1], or were newly created (new). “Jefferson” is the name of the company.

An ANOVA was performed with crisis type as predictor and actual change ratings of the chosen candidate as outcome variable. Crisis type had an effect, *F*(2,381) = 5.67; *p* = .004, η_p_^2^ = .03. Bonferroni corrected comparisons between the three conditions showed that actual change potential was more strongly associated with candidates chosen in the financial crisis (*M* = 6.08, *SD* = 0.86) than those chosen in the no crisis condition (*M* = 5.70, *SD* = 0.84; *p* = .003, 95% CIs [0.11, 0.65]). All other effects were non-significant (*p*s > .145). Overall these findings suggest that a candidate chosen in the financial crisis was more highly rated for their capabilities and competences (actual change potential) than a candidate chosen in a no crisis context.

## Study 3

### Leadership behaviors and traits

Agentic behavior was measured with the same items as in Study 1 (for all companies α > .90; relational crisis: *M =* 5.84, *SD* = 1.22, financial crisis: *M =* 6.21, *SD* = 1.18, no crisis: *M =* 6.20, *SD* = 0.90).

### Leader suitability

The same items as in the first two studies were presented after participants had made their choice for the organizational context. We conducted an ANOVA with candidate gender, candidate gendered traits, and choice as between-participants factors. The only significant effect was choice, *F* (2, 373) = 9.31, *p* < .001, η_p_^2^ =.05. We followed up with a set of Bonferroni corrected comparisons. Compared to a no crisis choice (*M* = 5.74, *SD* = 1.01), participants evaluated the suitability higher when they matched the candidate with a financial (*M* = 6.23, *SD* = 0.65; *p* = .009) or relational crisis (*M* = 6.12, *SD* = 0.86, *p* < .001). No other main or interaction effects were significant (*p*s > .250). The observed higher suitability ratings of chosen candidates in crisis contexts compared to a no crisis context suggest that closer attention is given to a leader’s qualities for good organizational functioning in contexts that are likely difficult to manage.

## Bibliography

1. Kulich C, Iacoviello V, Lorenzi-Cioldi F. Solving the crisis: When agency is the preferred leadership for implementing change. Leadersh Q [Internet]. 2018 Apr;29(2):295–308. Available from: http://dx.doi.org/10.1016/j.leaqua.2017.05.003

2. Kulich C, Lorenzi-Cioldi F, Iacoviello V, Faniko K, Ryan MK. Signaling change during a crisis: Refining conditions for the glass cliff. J Exp Soc Psychol [Internet]. 2015 Nov;61:96–103. Available from: https://linkinghub.elsevier.com/retrieve/pii/S002210311500089X

3. Kulich C, Lorenzi-Cioldi F, Iacoviello V, Faniko K, Ryan MK. Signaling change during a crisis: Refining conditions for the glass cliff. J Exp Soc Psychol [Internet]. 2015;61:96–103. Available from: http://linkinghub.elsevier.com/retrieve/pii/S002210311500089X
