# Supplementary material for: Contextualizing the think crisis-think female stereotype in explaining the glass cliff: Gendered traits, gender, and type of crisis
Source: PLoS One. 2021 Mar 2;16(3):e0246576. doi: 10.1371/journal.pone.0246576 (PMC7924740; doi:10.1371/journal.pone.0246576)
Supplement: Attachment — Submitted filename: PONE Comments to Author.docx [file pone.0246576.s004.docx]

I'd like the thank the authors for responding thoroughly to my arguments. In particular, I was impressed with the argument, in the paragraph starting on line 826, that gender and stereotypically gendered traits reflect different mechanisms. I only had a few minor comments:

Lines 394-395:

The authors wrote: "He was described with extreme, unmitigated agentic traits that included negative

content (e.g., authoritarian, tough, competitive; see [55]), aiming to disqualify him by this

unsympathetic description."

I'm not sure if the Helgeson (1994) reference cited here is very relevant. It talks about agency and communion in men and women and how it relates to their own well-being, and not how they are perceived by others, if I understood it correctly.Granted this was the least popular category, but I'm not sure if this is the best reference to support this.

Line 464:

The authors wrote: "The in H2 expected linear effect of..."

Typo?

Lines 562-566:

"In contrast, communal leadership traits may be deemed by participants as more likely inherited, attributed to internal characteristics, more stable, and therefore potentially more strongly associated to women than men, with a chosen leader less agile to adapt to changing situations.”

I'm wary about the perception that these traits are inherited. We speak about psychological traits, but would people think that something like communality is literally inherited? I think that it would be enough to say that communal traits are likely perceived to be internal and stable.

Lines 776-779:

The authors wrote: "For leadership behavior, path ‘a’ showed that the linear effect of relational crisis

versus no crisis versus financial crisis (C1) was related to more communal behavior (B =

0.40, SE = 0.08, p < .001, 95% CI [0.23, 0.56]), and more communal traits (B = 0.21, SE =

0.06, p < .001, 95% CI [0.09, 0.34]."

Would it be more correct to refer to preference for, or relevance of, communal behavior/traits, instead of "more" communal behavior/traits?

Lines 805-815

The authors didn't find support for a mediating effect on the relationship between crisis type and candidate gender preference. I had two comments. First, is it possible to see the actual results for the mediation, above knowing that it was not significant? Second, would it have been consistent with the authors' hypotheses to test for the mediating role of signalling change on the relationship between crisis type and preference for communal vs. agentic leader?

Lines 819-822:

The authors wrote: "However, while the financial crisis was the least preferred condition for communal candidates (consistent with H1), the no crisis condition was the least preferred for female candidates (consistent with H2)."

I don't have an issue with the info presented here, but I though that the wording was a bit awkward. In particular, I suspected following "However, while..." that the authors would present some contradictory findings, instead of stating that there hypotheses were supported.

Lines 990-996

It wasn't entirely clear what was being analyzed here. In particular, I had trouble understanding "an organizational context for which the importance of the communal leadership behavior was stronger compared to the match for agentic candidates". Is this the relational context?

But, overall, I had a very favorable impression of the revision and felt that it was much improved.
