# Supplementary material for: Contextualizing the think crisis-think female stereotype in explaining the glass cliff: Gendered traits, gender, and type of crisis
Source: PLoS One. 2021 Mar 2;16(3):e0246576. doi: 10.1371/journal.pone.0246576 (PMC7924740; doi:10.1371/journal.pone.0246576)
Supplement: Attachment — Submitted filename: Response to Reviewers.docx [file pone.0246576.s005.docx]

**Response to Reviewers**

EDITOR COMMENTS

1. **I would suggest taking out the term “linear” in the hypotheses and text. The authors were comparing three conditions which are categorical. Thus, to suggest that the differences between conditions were linear seem a distraction to me without evidence that could be used to substantiate the claim. For example, in testing H1, the percentages of participants to select communal leaders the three conditions are 50% (relational crisis), 38.5% (no crisis), and 27.2% (financial crisis). The differences between the conditions were not equivalent so I am not sure how you could say that this effect is linear (I suspect that the authors use the linear effects implicit assumption in regression models and treat the evidence as if it is actually linear). If the authors disagree, please provide results that compare linear effects with other types effects (e.g., curvilinear, cube, etc.).**

We can see your point, and we agree that the term “linear” may lead to confusion as we refer to three categorical conditions. In the literature theorising the Contrast analysis approach, a linear trend among three conditions does not actually make the claim that a straight line fits perfectly the relation between the independent and the dependent variables. A linear trend is tested by considering together the two orthogonal contrasts, in our case, the C1 and C2. The C1 alone tests only the difference between the two extreme conditions: for instance, between relational crisis versus financial crisis. The C2 (which is called  the “quadratic trend”), informs us whether the condition in-between is positioned in such a way as to more  closely represent a linear trend as opposed to a quadratic trend. In order to be able to conclude that the three conditions depict a linear trend, the C1 should be significant and the C2 should not. The C2 not being significant means that the in-between condition is not significantly distanced from the middle point between the extreme conditions, hence a trend that is close to a linear one. If C2 was significant, this would imply that the in-between condition was closer to one of the extreme conditions, meaning that the differences between the conditions are not equivalent. However, we recognise that for a reader not familiar with this approach and terminology, the term linear will be misleading. We thus followed your recommendation and  deleted, or replaced, the term “linear” in the entire manuscript (e.g. “In *Hypothesis 2*, which concerns gender, we thus also predicted a progressive pattern” (p12, line 263) ; “For communal leadership behavior, path ‘a’ showed a positive incremental effect of financial crisis - no crisis - relational crisis (C1) on communal behavior “ (p23, line 520)). We hope that this strategy has clarified our reports.

2. **Please include post-hoc comparison results in the tables so readers could read from the tables whether the numbers reported between either two conditions were significantly different.**

We do not report post-hoc comparisons in the article as this does not correspond to our analysis plan. The decision to use the Contrasts analysis approach was guided by two main points: the expected trend among our three conditions and the increased statistical power offered by contrast testing as opposed to omnibus tests and post-hoc comparisons. Thus, we powered our studies (in terms of sample sizes) based on this analysis plan. This means that any post-hoc comparisons will be underpowered. However, we do understand that the reader may be interested in having these analyses as well. We thus now provide two tables with post-doc comparisons in the Supporting information (Tables S1 Table and S3 Table).

3. **Please help readers understand why the roles expectations are important (imagine themselves to be leaders or employees) in the investigation of the glass cliff phenomenon and how this variation may have affect the hypothesized effects (or not). Are the candidates all expected to fill a leader’s role?**

Candidates in all three studies were expected to fill a leadership role.

As concerns participants, in Study 1 we manipulated their role, by asking them to think about themselves as a leader or an employee. In Studies 2 and 3 participants, who were employed themselves in real life, were asked to put themselves in the shoes of someone who needs to do the best for the company in terms of focusing on appropriate functioning. The exact formulation was: “You will then be asked to evaluate these candidates and choose the one who is best suited to ensure appropriate functioning of the company.” and then “Please select the candidate whom you consider to be the most suitable for this position at Jefferson's.” We believe that this instruction made people act in the interest of the company and not in their own interest (be they leaders or employees in real life).

We believe that glass cliffs should occur when people are being put in a decision maker’s position, which we now clearly state in the paper:

“As the recruitment for leadership positions is usually done by decision makers with managing functions in companies, rather than employees, the angle of the decision maker perspective is more likely to be source of glass cliffs decisions. Moreover, the potential motivations for glass cliff decisions discussed here also take the perspective of those in charge of hiring. Thus, in the present research we asked participants to focus primarily on this role.”  (p14, lines 320-325)

As we argue on pages 13-14, employees may have distinct motivations. However, we did not make specific hypotheses concerning the choice of a leader. This part was very explorative.

4. **Fig 1 on p. 22 was missing (only figure caption shown)**

We first added the figures to the revised manuscript but then PLOS ONE office told me to take the figures out again, so they are not included in the revised manuscript.

5. **Please revised the first sentence under the mediation analysis on p. 22. Shouldn’t it be communal behaviors rather than communal traits as the gendered traits did not have expected pattern in the above section.**

Thank you for pointing out this error. We have replaced traits by behavior.

6. **In studies 2 & 3, please specify the design before going details regarding the procedures. I am confused because the company performance (poor or strong) did not seem to be considered in the design.**

We moved the study design to the top of the Procedure sections for both Studies 2 (p28, line 633) and 3 (p40, line 904).

The no crisis condition is the “strong” and the two crisis conditions are the “poor” conditions and we realize that this has not been made explicitly clear to the reader. We now write in Study 2 “Company performance was presented as either poor or as strong (hereafter between brackets). Poor performance was presented for the two crisis conditions and strong performance for the no crisis condition.” (p28, lines 638-640)

7. **The information with regards to how participants got the manipulation check items wrong on p. 40 could be moved to the participant section when you specify who were excluded.**

Thank you. Indeed this part fits better in the participant section to which we have moved this part.

8. **Please provide the manipulation materials in the appendix.**

We created an appendix with all manipulation materials.

Please note: Doing an exact translation of the Spanish materials from Study 1 I got aware of some imprecisions which I corrected in the manuscript’s method section of Study 1.

**Reviewers' comments:**

**R2.1 If the odds ratio is noted at "eb", I would suggest the authors indicate this somehow as it is otherwise difficult to compare the power analysis to the results.**

**Confidence intervals were not consistently given for all results.**

We added a note in the article indicating that  “eb” represents the odd ratio (line 455).

We added CIs to all logistic regressions in Studies 1 and 2.

**R1.1. Lines 394-395:**

**The authors wrote: "He was described with extreme, unmitigated agentic traits that included negative content (e.g., authoritarian, tough, competitive; see [55]), aiming to disqualify him by this unsympathetic description."**

**I'm not sure if the Helgeson (1994) reference cited here is very relevant. It talks about agency and communion in men and women and how it relates to their own well-being, and not how they are perceived by others, if I understood it correctly. Granted this was the least popular category, but I'm not sure if this is the best reference to support this.**

Thank you for spotting this. We replaced the reference by Abele & Wojciszke (2007).

**R1.2. Line 464:**

**The authors wrote: "The in H2 expected linear effect of..." Typo?**

We rephrased as follows: “The effect of crisis type C1 that was expected in H2,...” (p21, line 471)

**R1.3. Lines 562-566: "In contrast, communal leadership traits may be deemed by participants as more likely inherited, attributed to internal characteristics, more stable, and therefore potentially more strongly associated to women than men, with a chosen leader less agile to adapt to changing situations.”**

**I'm wary about the perception that these traits are inherited. We speak about psychological traits, but would people think that something like communality is literally inherited? I think that it would be enough to say that communal traits are likely perceived to be internal and stable.**

Thank you for spotting this imprecision. We have rephrased this sentence as follows:

“In contrast, communal leadership traits may be attributed by participants to internal characteristics and considered as more stable, and therefore potentially more strongly associated to women than men, with a chosen leader less agile to adapt to changing situations. ” (p25, line 571)

**R1.4. Lines 776-779: The authors wrote: "For leadership behavior, path ‘a’ showed that the linear effect of relational crisis versus no crisis versus financial crisis (C1) was related to more communal behavior (B = 0.40, SE = 0.08, p < .001, 95% CI [0.23, 0.56]), and more communal traits (B = 0.21, SE = 0.06, p < .001, 95% CI [0.09, 0.34]."**

**Would it be more correct to refer to preference for, or relevance of, communal behavior/traits, instead of "more" communal behavior/traits?**

Thank you, we have corrected it to “For leadership behavior, path ‘a’ showed that the incremental effect of financial crisis - no crisis - relational crisis (C1) was related to higher relevance ratings of communal behavior (…)” (p35, 787)

**R1.5. Lines 805-815: The authors didn't find support for a mediating effect on the relationship between crisis type and candidate gender preference. I had two comments. First, is it possible to see the actual results for the mediation, above knowing that it was not significant? Second, would it have been consistent with the authors' hypotheses to test for the mediating role of signalling change on the relationship between crisis type and preference for communal vs. agentic leader?**

First: We have added the statistical indicators. “The mediation effect was not significant (*B* = - 0.01, *SE* = 0.03, 95% CI [-0.08, 0.06]). Thus, although signaling change was associated more with candidates chosen in both crisis contexts (path ‘a’: *B* = 0.36, *SE* = 0.04, *p* < .001, 95% CI [0.27, 0.44]), it was not specifically more associated with the choice of a woman (path ‘b’: *B* = -0.03, *SE* = 0.09, *p* = .775, 95% CI [-0.21, 0.16]).” (p36-37)

Second: No in the glass cliff literature the signaling hypothesis concerns the visible replacement of a male by a female (or a White by a Non-White) leader. When we look at trait quality this points more towards the actual qualities of a person, their way of leading, and this would be predicted to be explained by the relevance ratings of leader qualities (see mediation analysis in the paper).

We describe in this letter results if mediation was tested for signaling change for the candidate choice in terms of their gendered traits: The mediation effect was not significant (*B* = - 0.03, *SE* = 0.02, 95% CI [-0.07, 0.006]). Signaling change was associated more with candidates chosen in the relational than no crisis context (path ‘a’: effect of C1 *B* = -0.20, *SE* = 0.04, *p* < .001, 95% CI [-0.28, -0.12]; C2 which compares the no crisis to the two crisis contexts was also significant: *B* = -0.51, *SE* = 0.04, *p* < .001, 95% CI [-0.65, -0.37]). Signaling change was not associated with the choice of a communal candidate (path ‘b’: *B* = 0.16, *SE* = 0.10, *p* = .119, 95% CI [-0.04, 0.36]).”

**R1.6. Lines 819-822:The authors wrote: "However, while the financial crisis was the least preferred condition for communal candidates (consistent with H1), the no crisis condition was the least preferred for female candidates (consistent with H2)."**

**I don't have an issue with the info presented here, but I though that the wording was a bit awkward. In particular, I suspected following "However, while..." that the authors would present some contradictory findings, instead of stating that there hypotheses were supported.**

We did not intend to express contradiction, so we aligned it with your suggestion: “As expected, while the financial crisis was the least preferred condition for communal candidates (consistent with H1), the no crisis condition was the least preferred for female candidates (consistent with H2).” (p37, line 832)

**R1.7. Lines 990-996: It wasn't entirely clear what was being analyzed here. In particular, I had trouble understanding "an organizational context for which the importance of the communal leadership behavior was stronger compared to the match for agentic candidates". Is this the relational context?**

We rephrased the sentence to make it clearer. This part now reads:

“The perceived relevance of the communal behavior was higher for the type of crisis matched to the communal candidate (*M*= 5.82, *SE*= 0.10) compared to the one matched to the agentic candidate (*M* = 5.24, *SE* = 0.10).” (p45, line 1006)
